# Maximal Voluntary Ventilation Should Not Be Estimated From the Forced Expiratory Volume in the First Second in Healthy People and COPD Patients

**DOI:** 10.3389/fphys.2020.00537

**Published:** 2020-06-09

**Authors:** Matías Otto-Yáñez, Antônio José Sarmento da Nóbrega, Rodrigo Torres-Castro, Palomma Russelly Saldanha Araújo, Catharinne Angélica Carvalho de Farias, Armele de Fátima Dornelas De Andrade, Homero Puppo, Vanessa Regiane Resqueti, Guilherme Augusto de Freitas Fregonezi

**Affiliations:** ^1^Physical Therapy, Universidad Autónoma de Chile, Santiago, Chile; ^2^Programa de Doutorado em Biotecnologia RENORBIO, Universidade Federal do Rio Grande do Norte, Natal, Brazil; ^3^PneumoCardioVascular Lab/Hospital Universitário Onofre Lopes, Empresa Brazileira de Serviços Hospitalares (EBSERH), Universidade Federal do Rio Grande do Norte (UFRN), Natal, Brazil; ^4^Laboratório de Inovação Tecnológica em Reabilitação, Departamento de Fisioterapia, Universidade Federal do Rio Grande do Norte (UFRN), Natal, Brazil; ^5^Department of Physical Therapy, Faculty of Medicine, University of Chile, Santiago, Chile; ^6^Departamento de Fisioterapia, Universidade Federal do Pernambuco, Recife, Brazil

**Keywords:** maximal voluntary ventilation, forced expiratory volume in the first second, prediction formulas, prediction equation, COPD

## Abstract

**Purpose:**

To evaluate the concordance between the value of the actual maximum voluntary ventilation (MVV) and the estimated value by multiplying the forced expiratory volume in the first second (FEV_1_) and a different value established in the literature.

**Methods:**

A retrospective study was conducted with healthy subjects and patients with stable chronic obstructive pulmonary disease (COPD). Five prediction formulas MVV were used for the comparison with the MVV values. Agreement between MVV measured and MVV obtained from five prediction equations were studied. FEV_1_ values were used to estimate MVV. Correlation and agreement analysis of the values was performed in two groups using the Pearson test and the Bland–Altman method; these groups were one group with 207 healthy subjects and the second group with 83 patients diagnosed with COPD, respectively.

**Results:**

We recruited 207 healthy subjects (105 women, age 47 ± 17 years) and 83 COPD patients (age 66 ± 6 years; 29 GOLD II, 30 GOLD III, and 24 GOLD IV) for the study. All prediction equations presented a significant correlation with the MVV value (from 0.38 to 0.86, *p* < 0.05) except for the GOLD II subgroup, which had a poor agreement with measured MVV. In healthy subjects, the mean difference of the value of bias (and limits of agreement) varied between -3.9% (-32.8 to 24.9%), and 27% (-1.4 to 55.3%). In COPD patients, the mean difference of value of bias (and limits of agreement) varied between -4.4% (-49.4 to 40.6%), and 26.3% (-18.3 to 70.9%). The results were similar in the subgroup analysis.

**Conclusion:**

The equations to estimate the value of MVV present a good degree of correlation with the real value of MVV, but they also show a poor concordance. For this reason, we should not use the estimated results as a replacement for the real value of MVV.

## Introduction

The maximal voluntary ventilation (MVV) is the largest amount of air that a person can inhale and then exhale during a 12- to 15-s interval with maximal voluntary effort (Neder et al., 1999). This maneuver was used to provide information about the functioning of the inspiratory pump and chest wall and is used to evaluate maximum ventilatory capacity (Colwell and Bhatia, 2017) and respiratory muscle endurance, but the last ERS statement on respiratory muscle tests does not recommend its use for these purposes because mechanical aspects of the chest wall and lung tissue can affect the MVV value (Laveneziana et al., 2019). It is used for indirect calculation of the ventilatory reserve through a relationship with minute volume during a maximum exercise test (ATS/ACCP, 2003). The performance of this test can be modified by factors such as strength and endurance of the respiratory muscles and/or airways as well as chest wall biomechanics (Barreiro and Perillo, 2004; Pellegrino et al., 2005; Suh and Lee, 2017). From a technical point of view, the mobilized volume is extrapolated to the volume of air that would be moved in 60 s in order to avoid prolonged hyperventilation (Neufeld et al., 2018), and the result is expressed in liters/minute with an accuracy of ±10% (±15 L/min; ATS/ACCP, 2003).

Evaluation through this maneuver, together with other evaluations of lung function, was used for the follow-up of neuromuscular diseases (Rochester and Esau, 1994), and the prediction of the risk of postoperative complications (Bevacqua, 2015). Another important use is in cardiopulmonary exercise testing because it provides useful information for determining the ventilatory reserve (Ferrazza et al., 2009; Arena and Sietsema, 2011). The assessment of MVV is also used as a target for respiratory muscle training with normocapnic hyperpnea modality (Markov et al., 2001). In the past years, several equations have been described in the literature to estimate the MVV value, and the majority of these studies use the multiplication of the forced expiratory volume in the first second (FEV_1_) values by a constant (Cara, 1953; Gandevia and Hugh-Jones, 1957; Miller et al., 1959; Simonsson, 1963; Campbell, 1982). These predictive equations were developed to avoid the use of expensive equipment and patients’ intense ventilatory effort (Carter et al., 1987; Stein et al., 2003). Recently, the new publication of the Statement on Respiratory Muscle Testing of the European Respiratory Society (ERS) recommends estimating the MVV value as the FEV_1_ × 30 or 40 (Laveneziana et al., 2019). It is possible that these formulas were developed in a different historical context, where the availability of spirometry equipment that also evaluated MVV was scarce.

Additionally, the majority of the prediction formulas were developed by linear regression analysis and were validated based on good correlation values. However, the correlation coefficient is a measure of strength of the relationship between two variables and does not allow the evaluation of agreement nor accuracy between instruments (Carter et al., 1987). Thus, there is a lack of evidence based on concordance analysis between the values obtained from MVV and the estimated values obtained through the formulas.

In this context, the objective of this study was to evaluate the agreement between the direct MVV measure values and those obtained through equations based on FEV_1_ values in healthy people and chronic obstructive pulmonary disease (COPD) patients. Given the complexity of the respiratory system and the various factors that interact in the MVV test, we hypothesized that both direct and estimated values did not have an acceptable agreement in healthy people and COPD patients.

## Materials and Methods

A retrospective study was conducted with healthy subjects and patients with COPD. Data obtained from two previously conducted studies were analyzed (Araújo et al., 2012; Farias et al., 2014). These studies are approved by the Research Ethics Committee of Universidade Federal do Rio Grande do Norte (UFRN), Natal, RN, Brazil, under protocols 260/08 and 449/2010 (Brazilian Clinical Trials Registry RBR-7bqxm2) and done according to the Declaration of Helsinki of 1975. The evaluations of the healthy population were carried out between April 2009 and March 2010. COPD patients were evaluated between May 2011 and April 2012. Self-reported healthy subjects recruited from the university community with ages between 20 and 80 years, non-athletes, and with no history of smoking or pulmonary or neurological diseases composed the healthy group. The healthy ones with spirometric values below predicted (<80% of forced vital capacity, FVC, and/or FEV_1_, and below the lower limit of normality) were excluded. The patients with COPD were recruited in the respiratory outpatient clinic of the Onofre Lopes University Hospital (Natal, Brazil). Inclusion criteria were clinically stable patients, following the GOLD guidelines, with a post-bronchodilator spirometry value of FEV_1_/FVC less than 70%, PaO_2_ > 55 mmHg at rest with no recommendation for prescribing home oxygen therapy, and no other significant diseases that could prevent patient evaluation (Singh et al., 2019). Those with psychiatric disturbances, heart disease, or neurological or neuromuscular diseases associated were excluded. All patients gave informed consent.

### Measurements

Spirometry was used to perform the pulmonary function tests using a DATOSPIR-120 spirometer (SibelMed^®^, Barcelona, Spain) according to the recommendations of the ATS/ERS guidelines (Miller et al., 2005). Three technically acceptable and reproducible forced expiratory curves were obtained for each participant. Variability between them was <5%, and only the curve with the best performance was considered for analysis. The predictive reference values for the Brazilian population were calculated according to de Castro Pereira et al. (2007), and the FVC, FEV_1_, and FEV_1_/FVC in their absolute and relative values were considered for analysis. For the MVV, the participants were instructed to maximize ventilation by inhaling and exhaling as quickly and deeply as possible for 15 s (Miller et al., 2005), and values were expressed in liters per minute. The estimated MVV values based on the predictive formulas were determined by multiplying the FEV_1_, acquired during spirometry, by a constant (Table 1; Cara, 1953; Gandevia and Hugh-Jones, 1957; Miller et al., 1959; Simonsson, 1963; Campbell, 1982). Five equations were included. Two of the five included equations (Equations 2 and 3) are theoretical mathematical models, clinically not tested.

**TABLE 1:** Prediction equations.

Authors		Prediction equation
Simonsson, 1963	Equation 1	FEV_1_ × 30
Gandevia and Hugh-Jones, 1957	Equation 2	FEV_1_ × 35
Cara, 1953	Equation 3	FEV_1_ × 37.5
Campbell, 1982	Equation 4	FEV_1_ × 40
Miller et al., 1959	Equation 5	FEV_1_ × 41

*Abbreviations: FEV_1_, forced expiratory volume in the first second in liters.*

### Statistical Analysis

Data were expressed as mean ± SD, otherwise stated. Estimated MVV values were compared with the direct measure of MVV using Student’s *t* test for paired samples with a significance level of *p* < 0.05. Pearson coefficients of correlation were also performed between direct and estimated MVV values. The following classification was used to interpret the values found: negligible correlation (*r* < 0.10), weak correlation (*r* ≥ 0.1 to 0.39), moderate correlation (*r* ≥ 0.40 to 69), strong correlation (*r* ≥ 0.70 to 0.89), and very strong correlation (*r* = 0.90 to 1; Schober et al., 2018).

Agreements were evaluated using Bland–Altman plots (Bland and Altman, 1986), and the results were presented as bias (percentage of the difference between measured and estimated MVV values) and limits of agreement (± 1.96 SD). The 95% confidence intervals for both the bias and the limits of agreement were also added (Giavarina, 2015). Acceptable limits to the value of the equations would be accepted given a mean bias ≤5%, limits of agreement ≤10% (ATS/ACCP, 2003), and a 95% confidence interval of the mean bias within the line of equity of the Bland–Altman plot (i.e., 0% difference; Giavarina, 2015). Subgroup analysis was also conducted for healthy individuals (male and female) and for COPD patients (GOLD II, GOLD III, and GOLD IV).

Data were analyzed using GraphPad Prism 6 (GraphPad Software Inc., San Diego, CA, United States) software, and the level of significance was set at *p* < 0.05 with a two-tailed approach.

## Results

### Healthy Subjects

Data on 207 healthy people (47 ± 17 years, 102 male, and 105 female) were included. Anthropometric characteristics, spirometry, and data from MVV are shown in Table 2. For Student’s *t* test, only Equation 4 showed no significant differences with the direct measured MVV value. Regarding subgroup analysis, measured MVV values were not statistically different from Equations 4 and 5 in males. All equations were statistically different in females (Table 3).

**TABLE 2:** Anthropometric and spirometric values of healthy and COPD subjects.

	Healthy			COPD			
	All	Male	Female	All	GOLD II	GOLD III	GOLD IV
Subjects_(__n)_	207	102	105	83	29	30	24
Age_(years)_	47 ± 16.9	46.3 ± 16.4	47.7 ± 17.3	65.5 ± 6.4	65.4 ± 6.2	65.4 ± 6.6	65.6 ± 6.7
Weight_(kg)_	70.1 ± 12.3	77.3 ± 10.8	63.0 ± 9.1	71.5 ± 11.7	75.6 ± 13.0	69.8 ± 11.0	68.6 ± 10.0
Height_(__m)_	1.66 ± 0.93	1.72 ± 0.80	1.60 ± 0.67	1.65 ± 0.69	1.64 ± 0.85	1.65 ± 0.67	1.66 ± 4.6
BMI_(kg__/__m2)_	25.3 ± 3.6	26.0 ± 3.7	24.5 ± 3.4	26.2 ± 3.9	28 ± 4.2	25.2 ± 3.3	24.9 ± 3.4
FVC_(__L)_	3.8 ± 1.0	4.4 ± 0.8	3.1 ± 0.6	2.6 ± 0.7	2.8 ± 0.7	2.5 ± 0.6	2.3 ± 0.6
FVC_(%pred)_	95.3 ± 10.7	94.3 ± 10.1	96.0 ± 11.3	64.6 ± 15.1	75.9 ± 12.4	61.3 ± 12.7	55.1 ± 12.6
FEV_1 (L)_	3.2 ± 0.8	3.7 ± 0.7	2.7 ± 0.6	1.2 ± 0.4	1.6 ± 0.3	1.1 ± 0.2	0.7 ± 0.1
FEVl_(%pred)_	99.4 ± 11.3	97.9 ± 11.4	100.9 ± 10.9	41.1 ± 17.9	60.9 ± 12.5	37.1 ± 6.6	22.3 ± 3.9
FEV_1_/FVC_(__L)_	0.85 ± 0.06	0.84 ± 0.06	0.86 ± 0.06	0.46 ± 0.14	0.59 ± 0.09	0.46 ± 0.10	0.30 ± 0.06
MVV_(L/min)_	126.4 ± 36.6	149.4 ± 33.3	104.3 ± 23.6	46.5 ± 18.2	63.9 ± 13.6	45.0 ± 10.7	27.3 ± 6.7
MIP_(cmH20)_	101.5 ± 26.4	114.0 ± 28.1	89.4 ± 17.7	70.7 ± 18.9	72.5 ± 22.4	71.4 ± 18.6	67.8 ± 15
MEP_(cmH20)_	134.3 ± 42.5	160.2 ± 42.5	109.3 ± 23.5	134.6 ± 43.2	120.6 ± 46.6	140.6 ± 47.5	143.9 ± 27.9

*Data presented as mean and standard deviation. FVC, Forced Vital Capacity; FEV_1_, Forced expiratory volume in the 1st second; FEV_1_/FVC, Ratio of forced expiratory volume in the first second to forced vital capacity; n, number of subjects; m, meters; kg, kilograms; L, Liters; %pred, Percentage of predicted; MVV, Maximal Voluntary Ventilation; MIP, Maximal Inspiratory Pressure; and MEP, Maximal Expiratory Pressure.*

**TABLE 3:** Mean MVV values measured directly and predicted MVV values.

	Healthy			COPD			
	All	Male	Female	All	GOLD II	GOLD III	GOLD IV
Subjects_(__n)_	207	102	105	83	29	30	24
MVV_(L/min)_	126.4 ± 36.6	149.4 ± 33.3	104.3 ± 23.6	46.5 ± 18.2	63.9 ± 13.6	45.0 ± 10.7	27.3 ± 6.7
Equation 1_(L/min)_	95.2 ± 23.9**	110.0 ± 20.2**	81.1 ± 17.9**	35.2 ± 13.0**	49.1 ± 8.4**	33.5 ± 5.6**	20.8 ± 4.2**
Equation 2_(L/min)_	111.1 ± 27.9**	128.3 ± 23.6**	94.6 ± 20.8**	41.1 ± 15.2**	57.2 ± 9.8*	39.1 ± 6.5*	24.2 ± 4.9*
Equation 3_(L/min)_	119.1 ± 29.9**	137.4 ± 25.3**	101.4 ± 22.3*	**44.1 ± 16.3**	**61.3 ± 10.5**	**41.9 ± 6.9**	**26.0 ± 5.2**
Equation 4_(L/min)_	**127.0 ± 31.8**	**146.6 ± 27.0**	108.2 ± 23.8*	**47.0 ± 17.4**	**65.4 ± 11.2**	**44.6 ± 7.4**	**27.7 ± 5.6**
Equation 5_(L/min)_	130.1 ± 32.7*	**150.3 ± 27.6**	110.9 ± 24.4**	**48.2 ± 17.8**	**67.0 ± 11.5**	**45.8 ± 7.6**	**28.4 ± 5.7**

Results of direct maximal voluntary ventilation (MVV) measured and estimated by predicted equations. Student’s *t*-test for paired samples with a significance level of *P* < *0.05 were performed for the comparisons between measured MVV and estimated MVV.* *Significantly difference (*p* < 0.05) when the direct values were compared with the predictive values **Significantly difference (*p* < 0.0001) when the direct values were compared with the predictive values. The results expressed in bold letters correspond to the equations that did not show significant differences with the direct value of MVV. Data are presented are liter per minute (L/min).

**FIGURE 1:**
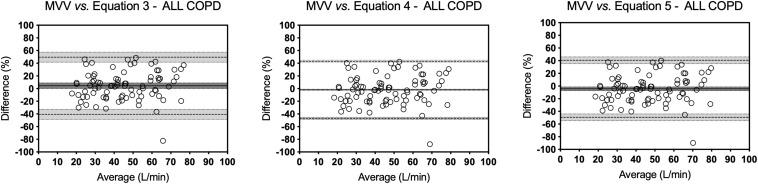
Bland–Altman analysis in Healthy group. Bland–Altman plots of those equations that presented a mean bias of ≤5% between MVV values measured directly and estimated for the healthy people. Bias is the average of the differences in percentage. Upper and lower limits of agreement are mean bias ±1.96 times its SD. The continuous lines represent the bias value, the dashed lines represent the limits of agreement, and the dotted lines represent the confidence intervals.

The results of all equations were significantly correlated with the measured MVV values (all *r*s = 0.86, *p*s < 0.0001). Similar results were also found for both male (*r* = 0.75, *p* < 0.0001) and female (*r* = 0.82, *p* < 0.0001) subgroups. As shown in Table 4, the mean bias of all equations varied from –3.9% (Equation 5) to 27% (Equation 1), and only Equations 3–5 presented a mean bias ≤5%. For males, this variation was between –1.7% and 29.1% and, for females, between –6.2 and 24.7% (Figure 1).

**TABLE 4:** Bland–Altman analysis between measured MVV and prediction equations.

	Healthy			COPD			
	All	Male	Female	All	GOLD II	GOLD III	GOLD IV
Equation 1 (%)	27.0 (-1.4 to 55.3)	29.1 (-1.3 to 59.5)	24.7 (-1.6 to 51.1)	26.3 (-18.3 to 70.9)	25.0 (-25.9 to 75.9)	27.8 (-12.5 to 68.2)	25.9 (-17.2 to 68.9)
Equation 2 (%)	11.7 (-17.4 to 40.8)	14.0 (-16.8 to 44.8)	9.5 (-17.2 to 36.2)	11.2 (-33.8 to 56.2)	10.0 (-41.0 to 61.0)	12.8 (-28.4 to 53.9)	10.8 (-33.0 to 54.5)
Equation 3 (%)	4.9 (-24.3 to 34.0)	7.2 (-23.8 to 38.1)	2.6 (-24.1 to 29.4)	4.4 (-40.7 to 49.5)	3.2 (-47.6. to 54.0)	5.9 (-35.4 to 47.3)	4.0 (-39.9 to 47.9)
Equation 4 (%)	-1.6 (-30.7 to 27.6)	0.8 (-30.2 to 31.7)	-3.8 (-30.6 to 23.0)	-1.9 (-47.0 to 43.1)	-3.2 (-53.7 to 47.4)	-0.5 (-41.9 to 41.0)	-2.4 (-46.3 to 41.6)
Equation 5 (%)	-3.9 (-32.8 to 24.9)	-1.7 (-32.6 to 29.2)	-6.2 (20.5 to -33.0)	-4.4 (-49.4 to 40.6)	-5.6 (-56.0 to 44.8)	-2.9 (-44.4 to 38.6)	-0.8 (-48.8 to 39.1)

*Bland-Altman between direct maximal voluntary ventilation (MVV) measured and equations 1 to 5. Agreement analyses between estimated MVV and measured MVV obtained in all 5 equations studied. Bias is the average of the differences ± SD between the 2 assay methods; upper and lower limits of agreement are mean bias ± 1.96 times its SD. Data presented as value of bias (and limits of agreement).*

### COPD Patients

Data of 83 COPD patients (65.5 ± 6.4 years, 29 GOLD II, 30 GOLD III, and 24 GOLD IV) were included. Equations 3–5 showed no significant differences from measured MVV values (Table 3). All equations were also significantly correlated with measured MVV values (all *r*s = 0.76, *p*s < 0.0001). When analyzing subgroups, significant correlations were found only for GOLD III (*r* = 0.38, *p* = 0.04), and GOLD IV (*r* = 0.49, *p* = 0.02).

**FIGURE 2:**
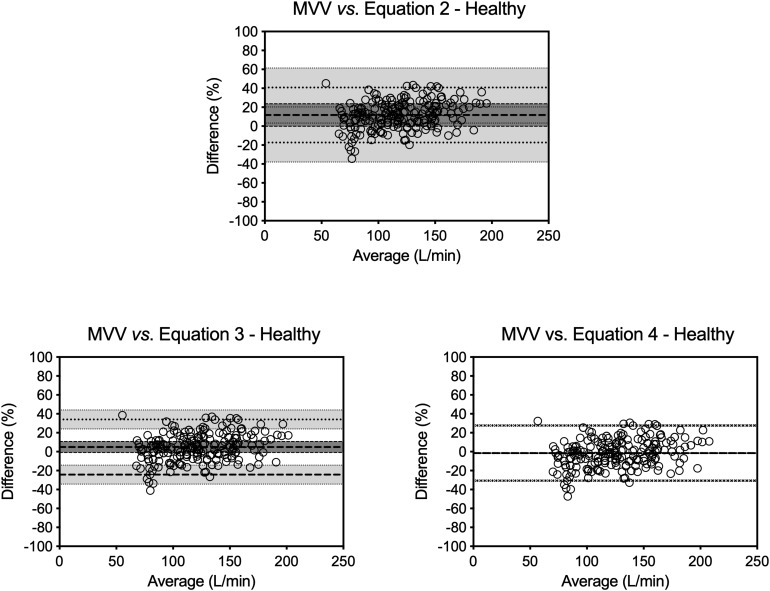
Bland–Altman analysis in COPD group. Bland–Altman plots of those equations that presented a mean bias of ≤5 between MVV values measured directly and estimated for the COPD patients. Bias is the average of the differences in percentage. Upper and lower limits of agreement are mean bias ±1.96 times its SD. The continuous lines represent the bias value, the dashed lines represent the limits of agreement, and the dotted lines represent the confidence intervals.

Poor agreements were also found between measured MVV values and those predicted from equations. For all patients, the mean bias varied from –4.4% (Equation 5) to 26.3% (Equation 1; Table 4). Despite Equations 3–5 presenting a mean bias of ≤5%, the limits of agreement were always greater than 40% (Figure 2).

## Discussion

Several studies use the estimation of MVV value from a prediction equation with the FEV_1_ value, usually multiplying the FEV_1_ by 35 or 40 (Callens et al., 2009; Werkman et al., 2011; Stevens et al., 2013). The main finding of this study was that, apart from strong correlations, a poor concordance was observed between measured MVV values and those estimated from equations. Although most of the formulas have statistically significant correlations, it is not possible to establish that both evaluation methods are equivalent through these statistical tests. When analyzing Bland–Altman plots, a poor agreement was observed. In the case of healthy subjects, the mean bias of all equations varied from –3.9% (Equation 5) to 27% (Equation 1), and only Equations 3–5 presented a mean bias ≤5%. For males, this variation was between –1.7 and 29.1% and, for females, between –6.2 and 24.7%. Nevertheless, as observed in Figure 1, the prediction equations not only overestimated (Equation 3) or underestimated (Equations 4 and 5) the measured MVV values, but also the limits of agreement were greater than that 10% recommended by scientific societies (Miller et al., 2005). All the equations presented a poor agreement. The limit of agreement analysis revealed a wide variation among equations. Although mean differences (bias) of Equations 2 and 3 in healthy individuals may be within the limits of acceptability of the test, its limits of agreement present a large dispersion, which does not allow validating the estimated value of MVV as a real value. These equations are based on a linear mathematical model, but possibly, the behavior of the respiratory system does not respond to a linear model. Therefore, it is complex to predict real physiological values using prediction formulas based on linear mathematical models.

We have found a wide average discrepancy between methods. This important discrepancy between the real and the estimated value could generate an underestimation or overestimation when an assessment test or isocapnic training about a percentage of the MVV value is established. Also, some formulas have differences close to 30% compared to the real value, which could generate important errors in the clinical interpretation if we only estimate the MVV value. The limits of the agreement and the bias value are quite wide, so it is not possible to establish that both methods are equivalent. There is no clear trend regarding the behavior of differences with the different equations. The dispersion of the points was always greater than the acceptable validity limits for this test.

On the other hand, these equations include parameters as FEV_1_ in healthy subjects, but the patients with chronic respiratory diseases may have abnormalities in the pulmonary function test that may change the accuracy of the measured MVV. Additionally, we analyze the equations in COPD patients to explore if the agreement presents the same behavior in both normal and pathological conditions.

The behavior was the same; poor agreements were also found between the measured MVV values and the ones predicted from equations. The mean bias varied from -4.4% (Equation 5) to 26.3% (Equation 1) with the greater variation observed in the GOLD III subgroup (from -2.9.0% with Equation 5 to 27.8% with Equation 1). Despite Equations 3–5 presenting a mean bias of ≤5%, the limits of agreement were always greater than 40%.

Maximal voluntary ventilation has poor specificity, is highly effort dependent, and can be uncomfortable for the patients. MVV depends on motivation and can be tiring for some patients (Laveneziana et al., 2019). It is reported that MVV depends on inspiratory and expiratory breathing effort in all type of subjects. The inspiratory airflow depends mainly on inspiratory muscle power in overcoming static elastic recoil of the respiratory system and resistive forces of the lung, and the expiratory airflow relies mainly on lung recoil (Lavietes et al., 1979; Milic-Emili and Orzalesi, 1998). Respiratory work is affected by respiratory rate, presenting a decrease in tidal volume and breathing power as the respiratory rate increases, and expiratory muscles have less time to harness the potential chemical energy for their action. This could affect the validity of the MVV estimation by means of equations because the expiratory technique during spirometry differs greatly from how it is performed in the MVV (Milic-Emili and Orzalesi, 1998). In normal subjects, lung recoil is known to be the major determinant of expiratory airflow in MVV performance. The use of equations of prediction based on FEV_1_ fails to take into account some physical characteristics that influence MVV (Neufeld et al., 2018), such as height, sex, and age. The literature has shown that individuals who smoke or are pregnant and people with cystic fibrosis had MVV values that deviate from sex, height, and age-matched controls (Stein et al., 2003; Hasan et al., 2013; Tell et al., 2014; Neufeld et al., 2018). The MVV execution involves repeated, rapid, and maximum ventilation, generating an increase in inspiratory and expiratory volumes in comparison with the tidal volume. COPD patients frequently present a phenomenon of hyperinflation, which generates a progressive decrease in inspiratory capacity (Gagnon et al., 2014). MVV is an assessment test that could be affected by hyperinflation, and this is the principal reason why we argue that it’s impossible to estimate the MVV value through a single expiratory parameter as the FEV_1_. This can be confirmed by the evidence of increases in the MVV value after lung volume reduction surgery in COPD patients (Benditt et al., 1997; Martinez et al., 1997).

Our results are in line with Nunes et al. (2016), who carried out a concordance study between the measured and estimated MVV value in 119 patients with pulmonary hypertension. The result showed that there was an overestimation of estimated values of lower measured MVV and underestimation at higher values. These findings confirm that it is not possible to predict the MVV value only through a multiple of the FEV_1_ value. This study only analyzes healthy subjects and COPD patients, so it is relevant to evaluate the concordance of these formulas in pathologies that present a restrictive ventilatory alteration. Our results confirm that, in order to know the value of the MVV, it is necessary to evaluate it not using a formula with the FEV_1_ value. The time when the spirometry teams did not evaluate the MVV is behind, and practically, most of the spirometry equipment allows this ventilatory test. The measurement of the MVV is considered an easily realizable test, and it is currently possible to perform it with most of the equipment available in the market, so it would not be advisable to replace its value by an estimate from the value of FEV_1_.

The MVV assessment provided complementary information and has clinical implications not only in healthy subjects and obstructive patients, but also in patients with restrictive diseases, as in the case of neuromuscular disease, given that they also perform MVV in the midrange of vital capacity. In this sense, MVV reflects the efficiency of lung recoil. The breathing pattern has a wide range of irregularities during the entire breathing period, and the calculation can conduct a mistake (Suh et al., 2019).

On the other hand, there are assessments that use the MVV, for example, the analysis of cardiopulmonary exercise testing, a routine evaluation of physical capacity. This outcome is useful for measuring the ventilatory reserve in patients with respiratory and cardiovascular disease (Guazzi et al., 2017). Taking this into account, the healthcare professional can distinguish between a cardiovascular and respiratory profile in the case of exercise intolerance (Nathan et al., 2019). However, we need to know the ventilatory reserve for this analysis, and the calculation of MVV provides an error risk.

In spite of having good levels of correlation and that some do not present significant differences with the real value of the MVV, when evaluating the agreement of these values, it is shown that it is not possible to consider these MVV evaluation formulas as valid due to presenting limits of agreement with a substantial dispersion.

Our study has some limitations. The number of patients diagnosed with COPD classified by GOLD categories is small. This only allows a global analysis of COPD patients that does not consider the severity of the disease. On the other hand, we did not analyze the hyperinflation. This parameter provides essential information because the efficiency of the movement of the diaphragm muscle depends on its correct biomechanical position, and the hyperinflation can influence the measure of MVV.

## Conclusion

In conclusion, MVV values estimated from equations are scattered and may underestimate or overestimate the real MVV value in healthy people and COPD patients. For this reason, a direct MVV measurement is suggested in this population instead of estimations through prediction equations. In consequence, we should not use the estimated results as a replacement for the real value of MVV.

## Data Availability Statement

The datasets generated for this study are available on request to the corresponding author.

## Ethics Statement

The studies involving human participants were reviewed and approved by Research Ethics Committee of Universidade Federal do Rio Grande do Norte (UFRN), Natal, RN, Brazil, under protocol 260/08 and 449/2010. The patients/participants provided their written informed consent to participate in this study.

## Author Contributions

GF, VR, CC, and AD contributed to design the study. PA, CC, and GF conducted assessments. MO-Y, AS, and GF analyzed, interpreted all experimental data, and were major contributors in writing the manuscript. All authors revised the manuscript.

## Conflict of Interest

The authors declare that the research was conducted in the absence of any commercial or financial relationships that could be construed as a potential conflict of interest.
